# PF4 at the Crossroads of Immunity and Neurodegeneration: A New Window Into Brain Aging

**DOI:** 10.1111/cns.70656

**Published:** 2025-11-19

**Authors:** Zili Xie

**Affiliations:** ^1^ Department of Dermatology, The Mark Lebwohl Center for Neuroinflammation and Sensation Icahn School of Medicine at Mount Sinai New York New York USA

Perioperative neurocognitive disorders (PND), encompassing postoperative delirium (POD), delayed neurocognitive recovery (dNCR), and postoperative neurocognitive disorder (PNCD), refer to neurocognitive impairments that occur during the perioperative period. They represent the most common complications of anesthesia and surgery in older patients. Currently, the most effective approaches for mitigating PND are nonpharmacological interventions, while pharmacological interventions remain limited, largely because the detailed mechanisms underlying the development of PND are still not fully understood. In a recent study published in *Molecular Psychiatry*, Dr. Lai et al. unveiled a compelling new perspective on our understanding of PND, identifying platelet factor 4 (PF4, also known as CXCL4) as a mechanistic bridge within a complement–platelet–brain signaling axis [1].

Β‐lactam antibiotics have been recognized for their neuroprotective and anti‐neuroinflammatory effects, and prior studies have shown that cefazolin can ameliorate postoperative cognitive dysfunction in young mouse models of PND. Based on this, the authors hypothesized that β‐lactam antibiotics could help prevent PND in elderly patients. They recruited a clinical cohort of 40 elderly patients and conducted a randomized, double‐blind clinical trial to evaluate the efficacy of perioperative β‐lactam antibiotics, including cefazolin and ceftriaxone, in preventing age‐related PND. Remarkably, patients in the ceftriaxone group exhibited a slower cognitive decline over time compared with those receiving cefazolin.

Neuroinflammation, characterized by glial activation and microglial accumulation in the hippocampus, is a key contributor to PND. The authors observed enhanced microglia–astrocyte cross talk mediated through the complement component 3 (C3)‐C3a receptor 1 (C3aR) axis. C3, a central component of the complement cascade, is a multidomain glycoprotein essential for immune function. Elevated C3 levels have been reported in the cerebrospinal fluid of older PNCD patients, and blocking C3aR has been shown to improve cognition after surgery in young PND mouse models. In this study, serum C3 levels were measured preoperatively. Both antibiotic groups showed postoperative elevations in C3, but the cefazolin group, exhibiting a higher incidence of mild PNCD, displayed greater C3 elevations than the ceftriaxone group. Importantly, elevated serum C3 levels significantly correlated with increased incidence of mild PNCD. To further establish causality, the authors used a selective C3 inhibitor (CR2‐Crry) and demonstrated that inhibition of C3 markedly reduced microglial infiltration, glial activation, and neuroinflammation after surgery. Similar protection was observed in *C3* ^*−/−*^ mice, confirming that C3/C3aR signaling critically mediates neuronal injury and cognitive decline in age‐related PND.

PF4, a cytokine released from activated platelets during coagulation, is well known for its interaction with heparin to form antigenic complexes that trigger IgG‐mediated immune responses in heparin‐induced thrombocytopenia. It has also been implicated in vaccine‐induced immune thrombotic thrombocytopenia [2, 3]. Beyond hematology, PF4 has recently gained attention in neuroscience and aging research. In a landmark study published in *Nature*, Villeda and colleagues demonstrated that PF4 suppresses microglial activation and the release of proinflammatory mediators, thereby mitigating neuroinflammation and improving learning and memory in aged mice [4]. These findings suggest that PF4 may confer neuroprotection and rejuvenate cognitive function in the aging brain.

In this study, serum PF4 levels were significantly higher in the ceftriaxone group than in the cefazolin group. Correlation analysis further revealed that higher PF4 levels were positively associated with better cognitive performance. To validate the mechanistic role of PF4, the authors utilized an aged mouse model of PND and demonstrated that exogenous PF4 administration rescued cognitive deficits even under persistent C3aR activation. These findings underscore PF4's potential as an immunomodulatory and neuroprotective agent. However, the mechanism of PF4's entry into the central nervous system (CNS) and its direct interactions with microglia or neurons remain unclear. Elucidating these pathways will be critical for understanding how peripheral PF4 exerts central neuroprotective effects.

This study delineates a novel molecular pathway underlying PND and provides a conceptual advance that bridges complement signaling, platelet biology, and neuroimmune regulation. If PF4 proves to be a common denominator across multiple aging‐associated brain disorders, it may represent not only a promising therapeutic target but also a molecular signature of resilience against neurodegeneration. Furthermore, this work challenges traditional neuron‐ or glia‐centric views of CNS diseases, suggesting that immune dysfunction may be a key driver of cognitive decline. This finding not only reinforces PF4's therapeutic potential but also raises important questions regarding its broader relevance across neurodegenerative and neuroinflammatory disorders.

## Conflicts of Interest

The author declares no conflicts of interest.

## Data Availability

Data sharing is not applicable to this article as no datasets were generated or analyzed during the current study.
